# Surface-active microrobots can propel through blood faster than inert microrobots

**DOI:** 10.1093/pnasnexus/pgae463

**Published:** 2024-10-15

**Authors:** Chenjun Wu, Toshihiro Omori, Takuji Ishikawa

**Affiliations:** Graduate School of Engineering, Tohoku University, Aramakiaza Aoba 6-6-01, Sendai, Miyagi 980-8579, Japan; Graduate School of Engineering, Tohoku University, Aramakiaza Aoba 6-6-01, Sendai, Miyagi 980-8579, Japan; Graduate School of Engineering, Tohoku University, Aramakiaza Aoba 6-6-01, Sendai, Miyagi 980-8579, Japan; Graduate School of Biomedical Engineering, Tohoku University, Aramakiaza Aoba 6-6-01, Sendai, Miyagi 980-8579, Japan

**Keywords:** microrobots, swimming, squirmer, red blood cells, Janus particle

## Abstract

Microrobots that can move through a network of blood vessels have promising medical applications. Blood contains a high volume fraction of blood cells, so in order for a microrobot to move through the blood, it must propel itself by rearranging the surrounding blood cells. However, swimming form effective for propulsion in blood is unknown. This study shows numerically that a surface-active microrobot, such as a squirmer, is more efficient in moving through blood than an inert microrobot. This is because the surface velocity of the microrobot steers the blood cells laterally, allowing them to propel themselves into the hole they are digging. When the microrobot size is comparable to a red blood cell or when the microrobot operates under a low Capillary number, the puller microrobot swims faster than the pusher microrobot. The trend reverses under considerably smaller microrobot sizes or high Capillary number scenarios. Additionally, the swimming speed is strongly dependent on the hematocrit and magnetic torque used to control the microrobot orientation. A comparative analysis between the squirmer and Janus squirmer models underscores the extensive applicability of the squirmer model. The obtained results provide new insight into the design of microrobots propelled efficiently through blood, paving the way for innovative medical applications.

**Significance Statement**

Microrobots stand at the forefront of transforming various medical procedures, from precision-targeted medication delivery to minimally invasive surgeries. Yet, these devices encounter substantial navigational challenges within the complex fluid environment of the human bloodstream. Our study bridges a critical gap by rigorously simulating the dynamics of surface-active microrobots within the blood. Through accurate simulations employing the boundary element method for fluid dynamics and the finite-element method for solid mechanics, our study sheds light on the intricate interplay between microrobots and red blood cells. It scrutinizes the effects of swimming mode, size disparity, Capillary number variations, magnetic torques, and hematocrit levels on navigation efficiency. Our findings lay the groundwork for future advancements in microrobotic applications in the blood circulatory system.

## Introduction

In the realm of biomedical engineering, navigating the intricate pathways of the human vascular system presents a significant challenge, particularly for the emerging field of microrobotics (1, 2). These microscale robots herald a new era of precision medicine, promising to revolutionize targeted therapeutic delivery and diagnostic procedures by navigating the bloodstream to access previously unreachable bodily sites (3, 4).

In addition to passive particles driven by external fields, synthetically powered microrobots like Janus particles demonstrate the potential for self-swimming within the bloodstream, powered by endogenous fuels like glucose (5, 6), urea (7, 8), ATP (9, 10), and triglycerides (11). Recently, near-infrared (NIR)-driven Janus particles have been examined in mice (12). Beyond these categories, bio-hybrid microrobots represent a burgeoning frontier. These systems integrate biological components like microorganisms, as their propulsion source (13). The dynamics of these biological propulsions can be effectively modeled using the squirmer model, capturing the complex fluid dynamics generated by the microorganisms they incorporate (14–16). Yet, the complexity of the blood environment, characterized by its high density of red blood cells (RBCs) and nonlinear flow dynamics, poses a formidable barrier to the efficient locomotion of these devices (17, 18). The behavior of individual RBC is already complex; however, multiple RBCs exhibit even more intricate behaviors (19), including rouleaux (20), collective-effects-induced tumbling to tank-treading transition (21), aligned-parachute and zigzag-slipper phases (22). Addressing this challenge necessitates a nuanced understanding of the propulsion mechanisms of microrobots within the vascular system, particularly focusing on the intricate dynamics between microrobots and RBCs. Such an understanding is critical for developing microrobots that can navigate blood vessels and move within organs with unprecedented precision, enhancing their potential for targeted delivery and diagnostic applications across a broad spectrum of medical conditions.

Despite impressive strides in microrobot locomotion, navigating the complex environments of the bloodstream remains a formidable challenge, as most previous studies predominantly conducted tests in highly controlled settings. Typically, microrobots have been tested for swimming in purified water (23), low-molecular solution (24), or simplified fluidic environments (5, 25). Furthermore, explorations within blood environments have seldom delved into interactions between microrobots and RBCs, leaving a critical area of study underexplored (26–28). A major difficulty arises from the traditional assumption of fluid homogeneity, which ceases to hold when the size of microrobots is akin to that of RBCs. In such cases, the conventional Stokes drag equation, which presumes a homogeneous fluid medium, fails to capture the discrete and heterogeneous nature of blood, necessitating a reevaluation of the fundamental principles governing microrobot locomotion in such biological environments.

In this work, we delve into the nuanced interactions between microrobots and the dense RBC environment of blood, a critical factor largely overlooked in prior studies. The coupling of the boundary element method (BEM) and finite-element method (FEM) allows for analyzing microrobot behavior in detail. BEM is utilized to model the fluid dynamics involving microrobots and RBCs, while FEM addresses the solid mechanics of RBC membranes. The accuracy of these methods has been verified by comparison and validation with blood experiments in our previous research (29–32), allowing a quantitatively accurate comparison of the microrobots’ performance. Our findings demonstrate that surface-active microrobots, such as squirmers, are moderately more efficient at navigating through blood than inert particles (also called inert microrobots). To quantitatively assess the behavior of microrobots in RBC surroundings, we provide a thorough analysis of how these interactions between microrobots and RBCs are influenced by swimming mode, microrobot size, applied magnetic torques, capillary number variations, and hematocrit levels. These results not only highlight the parameters crucial for optimal microrobot navigation in blood, but also lay the foundation for advances in the application of microrobotics in biomedical engineering, precision medicine, diagnostic technologies, and intelligent materials.

## Results

### Robot locomotion

**Fig. 1.**
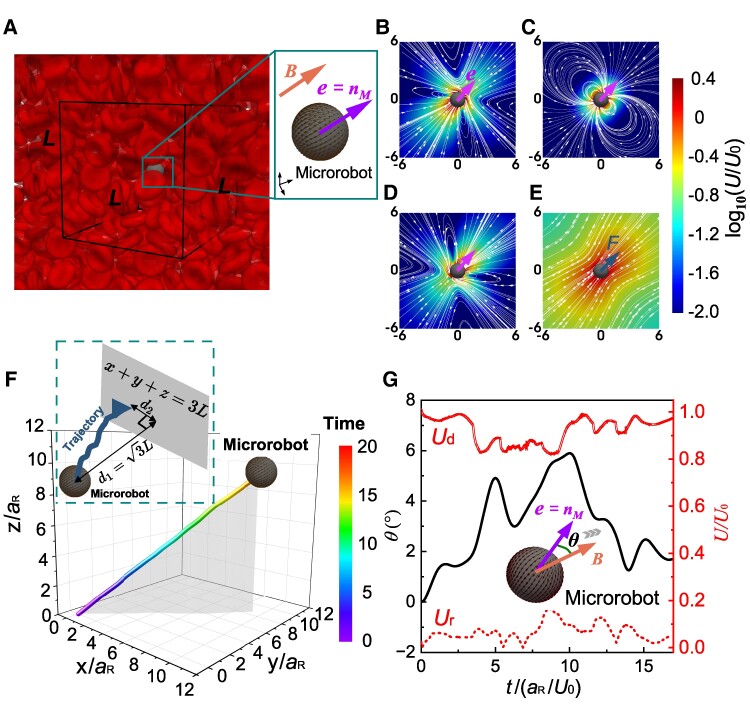
Numerical setup and locomotion of a microrobot. A) Schematic of a microrobot propelled through an RBC suspension. 44 RBCs are placed in a cubic main domain (black frame) with each side length defined as *L*. Enlarged view shows the detailed setting of the microrobot with surface velocity and magnetic field. The orientation (e) and magnetization direction ($n_{M}$) of the squirmer are identical, and the direction of the external magnetic field (B) is in the diagonal direction of the cubic domain. B–E) Flow-fields around four kinds of microrobots in an infinite domain (lab frame). Puller type microrobot with β=1 (B); Neutral type microrobot with β=0 (C); Pusher type microrobot with β=−1 (D); Force-driven microrobot (E). F and G) Locomotion characteristics of the neutral type microrobot propelled through an RBC suspension with ϕ=25%, Ca=0.5, and ϵ=0.8 under a dimensionless maximum magnetic torque $T_{m}^{*}$ of 10. Microrobot trajectory (F). Simulations are not terminated until the observed microrobot reaches the virtual plane of x+y+z=3L shown in the inset view. The diagonal ($U_{d}$) and perpendicular ($U_{r}$) velocity components of the microrobot, as well as the orientation and magnetization of the microrobot with respect to magnetic field (G).

We investigated microrobot locomotion in blood by employing two distinct microrobot models: the surface-active model, exemplified by squirmer retaining terms up to the second mode squirming (hereinafter referred to as squirmer) or Janus squirmer microrobots (15, 33), and the surface inert model, represented by a rigid sphere (34), as depicted in Figure 1A. A comprehensive list of microrobot types is provided in Table S1. The surface-active model propels itself by the surface squirming velocity, akin to the mechanisms observed in active colloids (35), droplets (36), and Janus particles (18, 37). Three distinct types of squirmers (Eq. 3) are employed in this simulation: puller, natural microrobot, and pusher. The velocity fields around them are shown in Fig. 1B, C, and D. The surface inert model presents a body-force-driven (hereinafter referred to as force-driven) microrobot (38). The velocity field generated by the force-driven microrobot is presented in Fig. 1E. We employed periodic boundary conditions in the simulations, within a cubic main domain with each side length defined as *L* (Fig. 1A). This allows us to model an effectively infinite fluid environment while maintaining computational feasibility. Since 98∼99% of formed elements of blood are RBCs (39), blood is assumed as a suspension of RBCs.

Initially, at t=0, a microrobot is placed at the origin of coordinate x(0)=(0,0,0) with orientation $e(0)=(1,1,1)/\sqrt{3}$. The unit vector ($n_{M}$) of magnetization M is always along the microrobot orientation, i.e. $e=n_{M}$. The magnetic field B is applied in the diagonal direction of the main domain, i.e. $B/|B|=(1,1,1)/\sqrt{3}$. The locomotion afterward is dynamically calculated until the microrobot reaches the plane of x+y+z=3L (*L* is the main domain size), at a distance $\sqrt{3}L$ apart from the origin, as depicted in the inset in Fig. 1F. We monitored the trajectory of a neutral squirmer-type microrobot (β=0) moving in an RBC suspension with hematocrit ϕ=25%, Capillary number representing RBC deformability Ca=0.5, and radius ratio ϵ=0.8 under a dimensionless maximum magnetic torque $T_{m}^{*}$ of 10, as illustrated in Fig. 1F. All nomenclature is detailed in Table 1. The definitions for *Ca*, ϵ, and $T_{m}^{*}$ are elaborated in Methods.

**Table 1.** Nomenclature in this study.

Parameter	Description	Value
*t*	Time	
*μ*	Viscosity of environmental Newtonian fluid	
$V_{R}$	Volume of an RBC	
$a_{R}$	Characteristic radius of an RBC	$\sqrt[{3}]{\frac{3{\text{V}}_{\text{R}}}{4\pi }}$
$G_{s}$	Shear elastic modulus of RBC membrane	
$n_{\mathrm{RBC}}$	Normal vector of the RBC plane	
$\theta_{c}$	Angle between $n_{\mathrm{RBC}}$ and *x*-axis	
*L*	Domain size	$9.03a_{R}∼12.27a_{R}$
*ϕ*	Hematocrit	10%∼25%
$a_{s}$	Microrobot radius	
$U_{d}$	Microrobot velocity component along the magnetic field (the diagonal direction of the main domain)	
$U_{r}$	Microrobot velocity component perpendicular to the magnetic field	
$U_{0}$	Speed of a solitary microrobot in an infinite Newtonian fluid without RBCs.	
*α*	Speed correction factor accounting for the robot–robot interaction	see Table 2
*β*	Swimming mode of a squirmer	−1.5∼1.5
e	Orientation of the squirmer microrobot	
*ψ*	Polar angle from the orientation vector e	$0^{\circ }∼180^{\circ }$
$v_{s}$	Tangential surface speed for the surface-active microrobot	
$d_{1}$	Microrobot displacement along the magnetic field (the diagonal direction of the main domain)	$\sqrt{3}L$
$d_{2}$	Microrobot lateral displacement (perpendicular to the magnetic field).	
M	Microrobot magnetization	
$n_{M}$	Magnetization direction	
B	Magnetic field	
$T^{*}$	Dimensionless magnetic torque	$\frac{4\pi |M||B|a_{s}}{3\mu U_{0}}\sin\;(\theta )n_{T}$
$n_{T}$	Unit vector associated with the magnetic torque	
*θ*	Angle between M, $n_{M}$, or e and B	
$T_{m}^{*}$	Maximum magnitude of dimensionless magnetic torque $\left(\frac{4\pi |M||B|a_{s}}{3\mu U_{0}}\right)$	1∼100
$C_{r}^{*}$	Relative resistance coefficient of the microrobot	$\alpha U_{0}/U_{d}$
*λ*	Lateral drift	$d_{2}/d_{1}$
*Ca*	Capillary number $\left(\frac{\mu U_{0}a_{s}}{G_{s}a_{R}}\right)$	0.2∼1.1
ϵ	Microrobot to RBC radius ratio ($a_{s}/a_{R}$)	0.2∼1.0
*C*	Area dilation modulus	10
$d_{G}$	Threshold distance for short-range repulsive force $F_{\mathrm{rep}}$	$0.15a_{R}$
$k_{c}$	Spring constant for short-range repulsive force $F_{\mathrm{rep}}$	$0.08G_{s}$
*Nd*	Normalized number density of RBCs around the microrobot	
$r_{\min}$	Position vector from the microrobot center to the closest point on the RBC^#^ surfaces	
$\theta_{R}$	Angle between $r_{\min}$ and e	$0^{\circ }∼180^{\circ }$

The microrobot navigates roughly along the magnetic field, i.e. diagonal to the main domain. The Supplementary Material, Movie S1 vividly illustrates the locomotion dynamics of the microrobot navigating through blood, capturing the intricate interactions with the surrounding RBCs. This visual representation highlights the complex interplay and the consequent deformations of RBCs, providing a comprehensive understanding of the microrobot’s maneuverability in a biological environment.

The velocity components of the microrobot are calculated in the direction of the magnetic field (along the diagonal of the main domain), denoted as $U_{d}$, and corresponding perpendicular direction $U_{r}$, as shown in Fig. 1G. We see that $U_{d}$ is much more obvious than $U_{r}$. In order to efficiently discuss the locomotion of a microrobot in the diagonal direction, we introduce the relative resistance coefficient of a microrobot $C_{r}^{*}$, defined as


(1) $$C_{r}^{*}=\frac{U_{d}^{\phi =0}}{U_{d}}=\frac{\alpha U_{0}}{U_{d}},$$

where $U_{d}^{\phi =0}$ is the velocity in the diagonal direction without RBCs. The coefficient *α* appears because of the triply periodic boundary condition, which can be derived analytically (40) and numerically (41). We explicitly show *α* values for force-driven microrobots in Table 2. For squirmer microrobots, robot–robot far-field interaction can be evaluated via stresslet, which decays rapidly with distance; therefore, *α* is close to 1.0 for any *ϕ*. The lateral drift of the microrobots (*λ*) is a measure of the directional control accuracy, defined as $\lambda =d_{2}/d_{1}$ (inset in Fig. 1F). $d_{1}=\sqrt{3}L$ (measured along B) and $d_{2}$ is the lateral displacement (measured perpendicular to B). The angle (*θ*) between the magnetic field and microrobot orientation aligned with magnetization during the simulation is shown in Fig. 1G. The medium deviation of *θ* still exists at a dimensionless maximum magnetic torque of $T_{m}^{*}=10$.

**Table 2.** Coefficient *α* in equation.

ϵ	*ϕ* (%)	*α*
1.0	25	0.6916
0.8	25	0.7517
0.6	25	0.8128
0.4	25	0.8748
0.2	25	0.9373
0.8	20	0.7691
0.8	15	0.7899
0.8	10	0.8161

The influence of hematocrit (*ϕ*) on microrobot locomotion is first detailed (cf. Fig. S1 and Supplementary Material, section S1). There, we observe that the relative resistance coefficient ($C_{r}^{*}$) generally increases with *ϕ*, while lateral drift (*λ*) exhibits variations. Additionally, the force-driven microrobot exhibits a markedly higher $C_{r}^{*}$ when compared with squirmer microrobots. The locomotion of these microrobots at a hematocrit level of 25% is dynamically illustrated in Supplementary Movie S2. In subsequent research, we consistently adopted the maximum hematocrit level of 25% for analysis, which is close to a physiological value in the microcirculation.

### Effect of magnetic torque

**Fig. 2.**
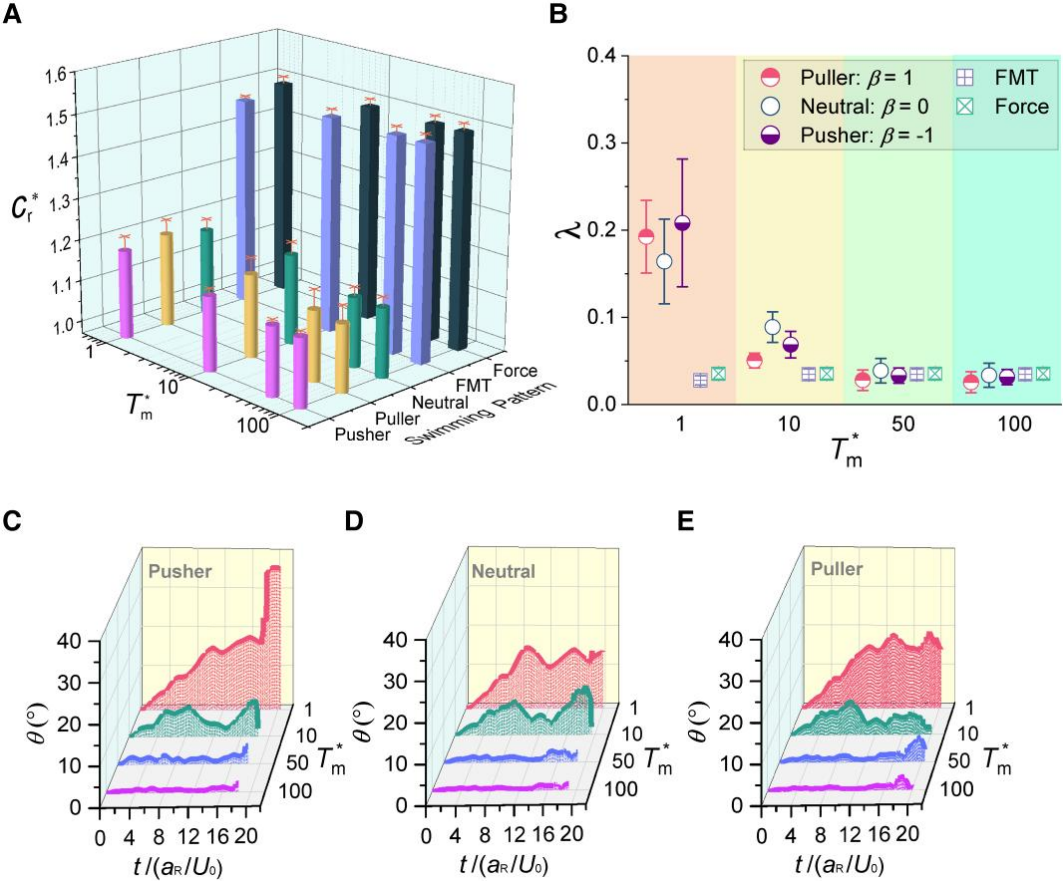
Effect of magnetic torque. The relative resistance coefficient A) and lateral drift B) of the various microrobots. *ϕ*, *Ca*, and ϵ are set to 25%, 0.5, and 0.8, respectively. Each data point for microrobot locomotion shows the means and standard errors of five independent trials. Angle (*θ*) between the external magnetic field and squirmer orientation (orientation of squirming velocity and magnetization) for the puller (β=1) C), neutral (β=0) D), and pusher (β=−1) E) microrobots. We performed five independent simulations, with each curve corresponding to the mean from five independent trials.

Of all the control and actuation methods, magnetic fields are consistently viewed as the most promising approach due to their exceptional controllability and deep penetration without tissue distortion, attenuation, or patient damage (42). The magnitude of dimensionless magnetic torque ($T_{m}^{*}$) given by Eq. 5 was tuned to explore the effect thereof on the resistance coefficient ($C_{r}^{*}$) and lateral drift (*λ*) of various microrobots. *Ca*, ϵ, and *ϕ* are held constant at 0.5, 0.8, and 25%, respectively. The results of $C_{r}^{*}$ are depicted in Fig. 2A. This investigation encompasses two distinct classes of force-driven microrobots: the conventional force-driven type, as delineated in preceding sections, and another class, the force-magnetic-torque-driven (FMT) microrobots, which employ a combined force propulsion and magnetic torque for orientation control identical to that of squirmer microrobots. To elaborate, the FMT microrobot is propelled by body forces identical to those used in conventional force-driven microrobots. Additionally, we have implemented an orientation vector (e) for the FMT microrobot, identical to that in the squirmer model. Magnetic torque is then applied to align this orientation vector with the external magnetic field (B), thus restricting the rotation of the FMT microrobot. This alignment mechanism, identical to that employed by squirmer microrobots, significantly reduces the rotational motion of the FMT microrobots. In contrast, conventional force-driven microrobots, which lack magnetic torque, can rotate freely. The FMT model is capable of emulating the behavior of microrobots made from hard magnetic materials in gradient magnetic fields and hybrid-driven microrobots, such as those operated by combined magnetic and acoustic fields (43).

Note that both force-driven and FMT microrobots are always subjected to more obvious $C_{r}^{*}$ than the squirmer-type microrobots. For the force-driven microrobot, $C_{r}^{*}$ is a constant across various $T_{m}^{*}$ since no magnetic torque is applied. In contrast, the application of magnetic torque to FMT microrobots triggers a slight fluctuation in $C_{r}^{*}$ and mildly reduces it, suggesting that magnetic torque alone is not sufficient to substantially improve the navigational efficiency of surface inert microrobots in blood. $C_{r}^{*}$ moderately declines for the squirmer microrobots, followed by a subtle drop when $T_{m}^{*}$ exceeds 10, indicating that a greater magnetic field or a material with higher remanence is beneficial to reduce resistance for the squirmer microrobots. This is because, under high $T_{m}^{*}$ conditions, the orientation of squirmer microrobots aligns almost parallel to the magnetic field, enabling them to navigate straighter toward the goal. Consequently, squirmer microrobots reach their destination faster.

To quantitatively assess this enhanced efficiency, we have computed the average velocity magnitude ⟨|U|⟩ of all microrobots, defined as $|U|=\sqrt{U_{d}^{2}+U_{r}^{2}}$ (Supplementary Material, section S2). As depicted in Fig. S2, Lower ⟨|U|⟩ are found in force-driven microrobots compared with squirmer microrobots, attributed to unique hydrodynamic properties of the squirmer microrobots. ⟨|U|⟩ remains relatively consistent across various magnetic torque levels ($T_{m}^{*}$) for each type of microrobot, including both squirmer and force-driven microrobots. This consistency further indicates that the application of magnetic torque reduces the resistance coefficient $C_{r}^{*}$ of the squirmer microrobots, primarily by enhancing their velocity along the magnetic field and simultaneously restraining their velocity perpendicular to the magnetic field. However, for the FMT microrobot, this magnetic-torque-induced constraint on perpendicular velocity is less pronounced.

Figure 2B illustrates the lateral drift (*λ*) of different microrobots as $T_{m}^{*}$ undergoes variation. Notably, both force-driven and FMT microrobots exhibit a nearly consistent *λ* across different $T_{m}^{*}$ values, indicating that partially restricted rotation has nearly no effect on *λ*. Moreover, their *λ* is markedly lower compared with that observed in squirmer-type microrobots. For squirmer microrobots, *λ* considerably declines when $T_{m}^{*}$ is below 10, followed by a moderate reduction from $T_{m}^{*}=10$ to 50, and eventually a basin when $T_{m}^{*}$ exceeds 50. A nearly tenfold decrease in *λ* is observed across the range of $T_{m}^{*}$ values. This suggests that higher magnetic torque can reduce *λ* for the squirmer-type microrobot.

Furthermore, we quantified the squirmer-type microrobot orientation by monitoring the angle (*θ*) between their orientation of squirming velocity and magnetization and the external magnetic field, as depicted in Fig. 2C–E. *θ* exhibits a drastic drop, followed by a moderate decrease, and eventually a slight decline, as $T_{m}^{*}$ increases. Contour profiles of *θ* curves vary from $T_{m}^{*}=1$ to $T_{m}^{*}=50$, and nearly keep the same when $T_{m}^{*}\geq 50$. We calculated their mean squared angular spread ($\left\langle \theta^{2}\right\rangle$) of squirmer microrobots under various values of $T_{m}^{*}$ (Supplementary Material, section S3 and Fig. S3). It is important to note that in our simulations, rotational diffusion is constrained by the presence of magnetic torques, which distinguishes these results from those of nonmagnetic microrobots (44, 45).

### Effect of radius ratio

**Fig. 3.**
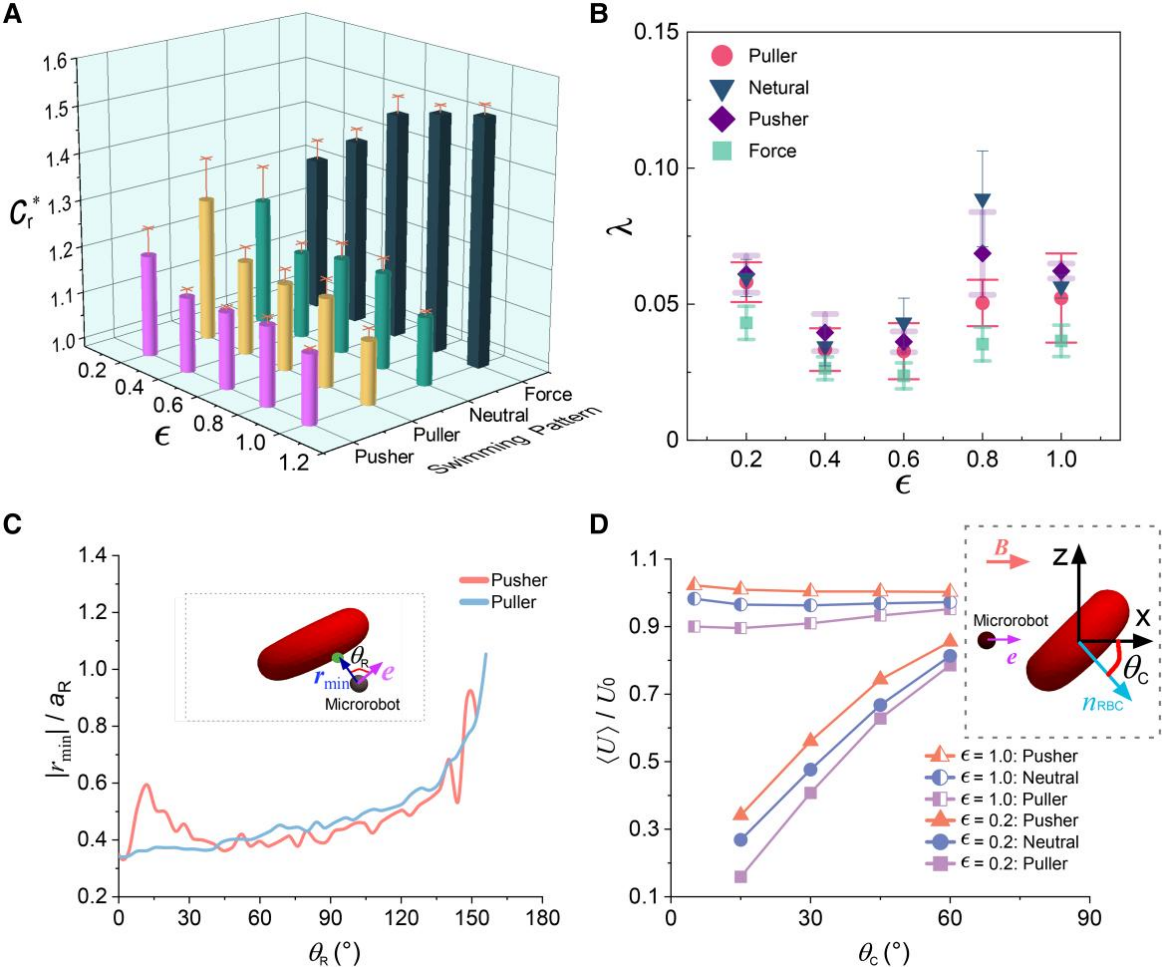
Effect of radius ratio. The relative resistance coefficient A) and lateral drift B) of the various microrobots (ϕ=25%, Ca=0.5, and $T_{m}^{*}=10$). Each data point for microrobot locomotion shows the means and standard errors of five independent trials. C) Minimum distance between the RBC surface and center of the puller (β=1) and pusher (β=−1) microrobots and the corresponding $\theta_{R}$ during simulations. Insert panel shows the definition of $\theta_{R}$. A green dot represents the nearest point on the RBC surface to the microrobots. e and $r_{\min}$ are the microrobot orientation and the vector from the microrobot center to the nearest point on the RBC surface, respectively. D) Velocity of the squirmer-type microrobots in the *x* direction under the influence of a solitary RBC, with varied ϵ. Insert panel illustrates the simulation setup, where the microrobot orientation e (same as the direction of magnetization M) are aligned along the *x*-axis initially. The external magnetic field B is always aligned along the *x*-axis. The angle ($\theta_{c}$) between the normal vector of the RBC plane ($n_{\mathrm{RBC}}$) and *x*-axis is tuned.

The size ratio between the microrobots and RBCs (ϵ), given by Eq. 6, largely affects microrobot locomotion. Therefore, we analyzed the effect of ϵ on the resistance coefficient ($C_{r}^{*}$) and lateral drift (*λ*) of various microrobots by changing only ϵ (Ca=0.5, ϕ=25%, and $T_{m}^{*}=10$). Figure 3A shows the dependence of $C_{r}^{*}$ on ϵ. As ϵ declines, $C_{r}^{*}$ of the squirmer-type microrobots distinctively rises, especially from ϵ=0.4 to 0.2. Remarkably, even at ϵ=0.2, $C_{r}^{*}$ for the puller microrobot remains lower than that of the force-driven microrobot, although it represents the highest value among the squirmer microrobots. Notably, at ϵ=0.2, the pusher microrobot underscores the obvious superiority in terms of $C_{r}^{*}$. However, at ϵ=1.0, an interesting convergence occurs. All squirmer microrobots exhibit nearly identical $C_{r}^{*}$ values. This suggests that under the influence of a large size ratio, the distinct characteristics observed at a low size ratio tend to converge, resulting in comparable resistance coefficients for all squirmer microrobots. This finding provides valuable insights into the profound impact of variations in ϵ on the hydrodynamics of microrobots, ultimately influencing their performance across various design configurations. Compared to the squirmer-type microrobots, the force-driven microrobot displays an opposite trend. Variation in ϵ triggered moderate fluctuations in *λ* for all microrobots, as shown in Fig. 3B. Force-driven microrobot exhibits less *λ* than the squirmer-type microrobots.

To investigate the mechanism underlying the disparity in $C_{r}^{*}$ under ϵ=0.2, we conducted calculations involving the closest point on the RBC surfaces to the microrobots. Vector $r_{\min}$ represents the position vector from the microrobot center to the closest point on the RBC surfaces, and $\theta_{R}$ denotes the angle between the microrobot orientation e and $r_{\min}$. The results are illustrated in Fig. 3C. The puller microrobot exhibits an approximate plateau for $\theta_{R}$ ranging from 0 to $40^{\circ }$, followed by a continuous increase. This phenomenon suggests that RBCs obstruct the path in front of the puller microrobot, leading to a significantly higher $C_{r}^{*}$. In contrast, for the pusher microrobot, $\left|r_{\min}\right|$ exhibits an initial wide peak, then reaches a plateau, and eventually shows an upward trend with enhanced $\theta_{R}$; this suggests that RBCs tend to accumulate on the sides rather than in front of the microrobot, which does not significantly enhance $C_{r}^{*}$. There is a notable difference between ϵ=0.2 and ϵ=1.0 for the squirmer-type microrobots. To delve deeper into this disparity, we conducted additional simulations involving a single RBC positioned in front of a microrobot. The schematic is depicted in the insert panel of Fig. 3D. The initial center distance between the RBC and microrobot was set to $1.8a_{R}$. The microrobot orientations were initially set along the *x*-axis, and the external magnetic field was maintained to the *x*-axis. The angle between the normal vector of the RBC plane and *x*-axis ($\theta_{c}$) was tuned. Figure 3D illustrates the average velocity ⟨U⟩ of the microrobots during the locomotion from the origin to the x=L plane, normalized by $U_{0}$, with varied ϵ. Microrobots with ϵ=0.2 show monotonically slowed velocity with declining $\theta_{c}$. The large microrobots with ϵ=1.0 exhibit negligible sensitivity to $\theta_{c}$ and swim significantly faster than the small microrobots with ϵ=0.2 (Movie S3). When $\theta_{c}$ equals $5^{\circ }$, the small microrobots took considerable time to reach the goal plane; hence, the results are discarded from the figure, though the large microrobots could easily escape from the RBC. Interestingly, the pusher microrobots exhibit larger velocity than puller microrobots in small-size scenario. This finding is consistent with the behavior of the small microrobots (ϵ=0.2) in RBC suspension. The primary factor contributing to the trend is the pronounced impact of the nearest RBC predominantly on small-sized microrobots.

### Effect of capillary number

**Fig. 4.**
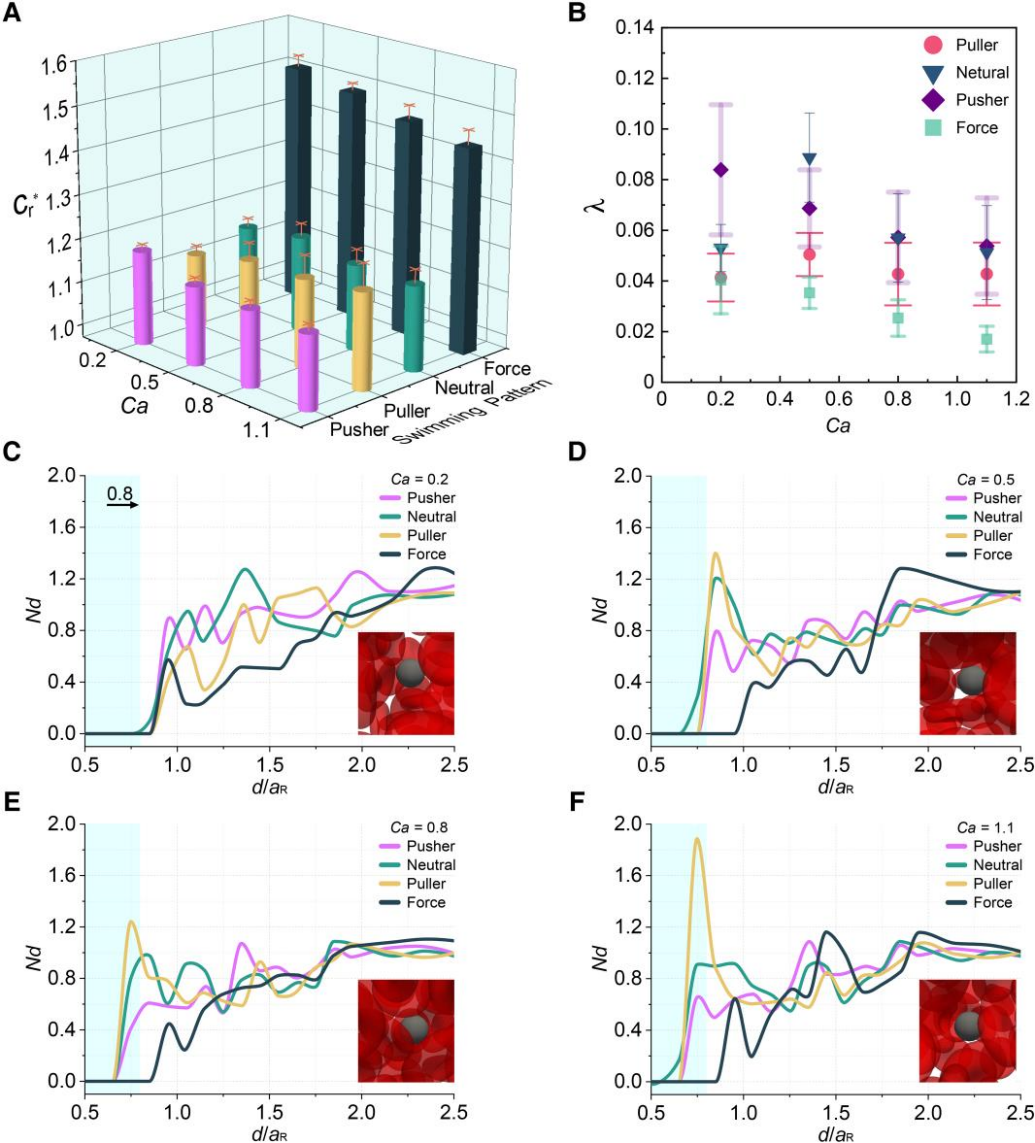
Effect of Capillary number (*Ca*). The relative resistance coefficient A) and lateral drift B) of the various microrobots. C–F) Corresponding normalized number density of RBCs (*Nd*) for the squirmer and force-driven microrobots under different *Ca* values: 0.2 (C), 0.5 (D), 0.8 (E), and 1.1 (F). The cyan area corresponds to the radius of the microrobots. The inset provides a detailed view of the puller microrobots and surrounding RBCs at the final observation time point. $T_{m}^{*}$, *ϕ*, and ϵ are held constant at 10, 25%, and 0.8, respectively. Each data point for microrobot locomotion shows the means and standard errors of five independent trials.

Capillary number *Ca* represents the ratio of viscous force to the elastic force exerted on an RBC, defined by Eq. 21. We investigated its effect on the relative resistance coefficient ($C_{r}^{*}$) and the lateral drift (*λ*) of the microrobots (ϕ=25%, ϵ=0.8 and $T_{m}^{*}=10$). *Ca* significantly affects RBC shape, deforming RBCs from discoidal shape as *Ca* is increased. In Fig. 4A, we provided $C_{r}^{*}$ under various *Ca*. All squirmer-type microrobots appear much lower $C_{r}^{*}$ than the force-driven microrobot. Force-driven, pusher (β=−1), and neutral (β=0) microrobots show increased $C_{r}^{*}$ with decreasing *Ca*. In contrast, $C_{r}^{*}$ of puller microrobot (β=1) decreases with decreasing *Ca*. These results illustrate that the squirmer-type microrobots are more efficient in moving through blood than the force-driven microrobot and the locomotion speed strongly depends on the squirmer type.

We consider the flow field around the microrobot to understand the difference in speed due to the squirmer type. A pusher microrobot slows down when it approaches nearby RBCs because the fluid pushed out in front is reflected by the RBCs (46). Meanwhile, RBCs are pushed away from the microrobot. Once the pusher microrobot has passed the RBCs, the fluid pushed out behind accelerates it. The neutral microrobot is a silent swimmer in that its far-field flow in the dilute regime decays as $∼1/d_{s}^{3}$, where $d_{s}$ is the distance between the microrobot center and an observation point. The puller microrobot pulls RBCs towards itself and undergoes acceleration when at a considerable distance from the RBCs, followed by a deceleration as the microrobot approaches RBCs. Once the microrobot passes the RBCs, the fluid drawn towards it hinders its locomotion.

At Ca=0.2, RBC deformation is very small, exerting higher resistance to the microrobots. This is evidenced by the barely discernible RBC deformation in the inset of Fig. 4C. The puller microrobots could utilize RBCs to speed them up, leading to relatively lower $C_{r}^{*}$ than the neutral microrobot. The latter cannot drain obstacle RBCs in the way. Higher *Ca* results in larger deformation of RBCs (illustrated in the insets of Fig. 4D and E), corresponding to lower $C_{r}^{*}$ for the force-driven, pusher, and neutral microrobots. RBCs exhibit parachute shape at Ca=1.1 (47), a phenomenon captured in the inset of Fig. 4F. The puller microrobot pulls RBCs in front of itself, and the RBCs with parachute shape extensively hinder its locomotion. However, the pusher microrobot drives the parachute-shaped RBCs away, and the neutral microrobot at least does not attract the RBCs. As a result, the pusher microrobot exhibits the lowest $C_{r}^{*}$ under Ca=1.1.

*λ* under various *Ca* is shown in Fig. 4B. Nearly all microrobots show lower *λ* with enhanced *Ca* except for the neutral microrobot. For the latter, variations in *Ca* triggered only slight fluctuations in *λ*; all values are around 0.06∼0.1, which is small considering the microrobot size of 0.8 and the travel distance of $\sqrt{3}L$. The force-driven microrobot unfolds lower *λ* than the squirmer-type microrobots under all *Ca* variations. This is because when the orientation of a self-locomotive microrobot is shifted, it is propelled in the shifted direction. These results imply that a force-driven microrobot can be considered under the strict limitation of *λ*, although the microrobot affords higher resistance.

To discuss robot–RBCs interactions, we calculate the normalized number density of RBCs around the microrobot. *Nd* is defined using the distance *d* from the microrobot as


(2) $$Nd(d,d+\Delta d)=\frac{1}{n_{0}}\frac{\Delta M}{\Delta v},$$

where $n_{0}$ is the average number density of RBCs in the suspension, ΔM is the ensemble-averaged number of RBCs in the region d∼d+Δd, and Δv in the volume of spherical shell defined in the range d∼d+Δd. Nd>1 indicates aggregation of RBCs, whereas Nd<1 indicates a decrease in RBCs. The results are illustrated in Fig. 4C–F. With increasing *Ca*, the nearest RBC appears at a smaller distance for the squirmer-type microrobots. Especially, the corresponding nonzero value appears under $d<0.8a_{\mathrm{RBC}}$ when Ca≥0.8, that is, RBC center is inside the microrobots, though it is not actually penetrated. The results further demonstrate that the RCB shape changed from a discoidal to a parachute shape. In contrast, for the force-driven microrobot, the first nonzero value does not emerge until $d>0.8a_{\mathrm{RBC}}$ under various *Ca*. For the puller microrobot (cf. Fig. 4F), the large and high first peak from the left is observed at Ca=1.1. This peak is more pronounced than the corresponding peaks of the other microrobots, unequivocally affirming the RBC accumulation around the puller microrobot under Ca=1.1. Consequently, the puller microrobot is in the trap composed of RBCs with a parachute shape. In contrast, when Ca=0.2 (Fig. 4C), the first peak from the left is noticeably lower and appears distant from the microrobot center when compared with the first peaks of the other microrobots, indicating that RBCs easily slip towards the side of the puller microrobot. The neutral and pusher microrobots unveil the opposite trend. The first peak does not grow in magnitude with increasing *Ca*, despite shifting towards the left. *Nd* for the force-driven microrobot does not significantly change under different values of *Ca*. We conclude that the puller microrobot is optimal under low *Ca* scenarios, such as during slow propulsion or in areas of reduced erythrocyte deformability due to lesions such as malaria (48), whereas the pusher microrobot is preferable under high *Ca* scenarios during fast propulsion.

When microrobots operate *in vivo*, they exert forces on surrounding RBCs, potentially leading to hemolysis. To assess this risk and better understand the mechanical effects of microrobots, we measured the time-average max isotropic membrane tension $\left\langle \tau_{p}^{\max}\right\rangle$ on RBC membranes to characterize their deformation under these conditions (49) (Supplementary Material, section S4). The results are shown in Fig. S4, which indicate that the puller microrobot exerted greater tension on the RBCs than the pusher microrobot.

### Comparative analysis of squirmer and Janus squirmer microrobot models

The squirmer model lays the groundwork for comprehending the propulsion dynamics across a broad spectrum of microrobots, typically simplifying the analysis by excluding squirming modes beyond the second (15). However, its capacity to fully encapsulate the intricate propulsion mechanisms of various microrobots warrants further exploration due to this omission. To address this, our analysis extends to a comprehensive examination of the applicability of the squirmer model across different microrobot propulsion systems. One interesting application is the Janus squirmer model (37), a specialized variant within the squirmer framework. Within this analysis, we maintain specific control variables to ensure consistency and accuracy. Specifically, *ϕ*, *Ca*, ϵ, and $T_{m}^{*}$, are kept constant at 25%, 0.5, 0.8, and 10 respectively. This comparative analysis focuses on discerning the unique propulsion characteristics of the Janus squirmer model against the squirmer model. Our goal is to uncover the nuances in propulsion strategies and dissect their hydrodynamic consequences. This investigation is crucial not only for deepening our theoretical understanding of microrobot dynamics but also for its practical implications in biomedical fields, where the precision and versatility of microrobot maneuverability are of utmost importance.

**Fig. 5.**
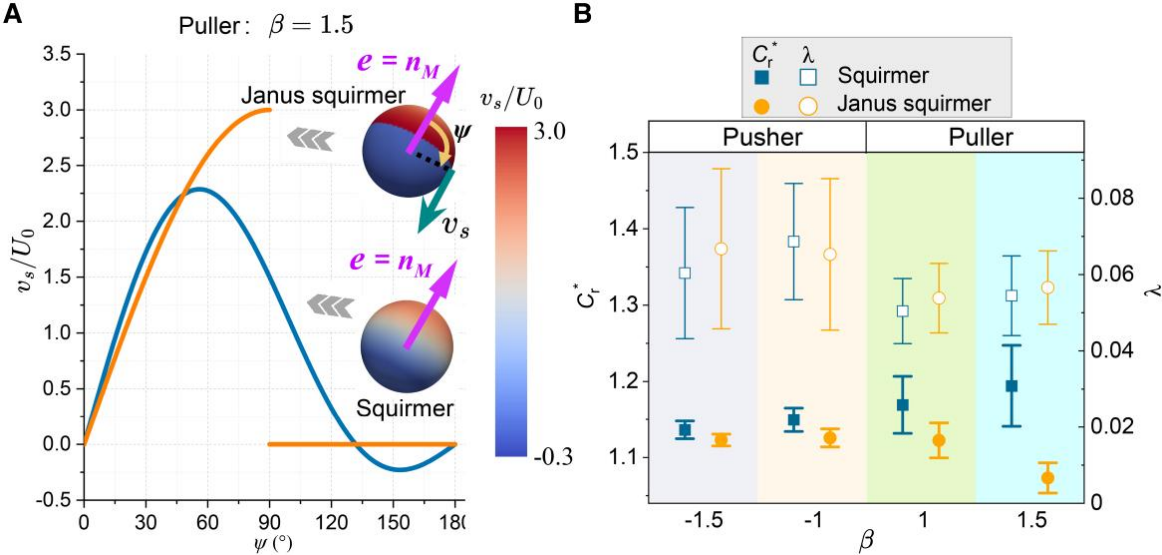
Comparative analysis of the squirmer and Janus squirmer models. A) The surface velocity profiles for Puller-type Janus squirmer and squirmer microrobots with β=1.5. The orientation (e) and magnetization direction ($n_{M}$) of the squirmer and Janus squirmer microrobots are identical. B) The relative resistance coefficient and lateral drift of the various microrobots under ϕ=25%, Ca=0.5, ϵ=0.8, and $T_{m}^{*}=10$. Each data point for microrobot locomotion shows the means and standard errors of five independent trials.

Janus squirmer microrobots are typically composed of colloidal spheres, each featuring a distinct active cap that envelops approximately half of their surface area (18, 37). This asymmetry in surface composition results in a unique surface slip velocity, a fundamental characteristic outlined in Eq. 4. To elucidate the distinctions in propulsion mechanics between the squirmer and Janus squirmer models, particularly in terms of slip velocity, we present a typical comparative representation (Puller with β=1.5) in Fig. 5A (see the other surface velocity profiles in Fig. S5). Janus squirmer microrobots exhibit discontinuous patterns of surface slip velocity on their equators, distinguishing them from their squirmer counterparts. It should be noted that the squirmer and Janus models both yield identical surface slip velocity patterns in the context of a neutral type (37).

Figure 5B depicts the relative resistance coefficient ($C_{r}^{*}$) and lateral drift (*λ*) of the puller and pusher microrobots. Both pusher and puller microrobots characterized by the Janus model show consistently lower $C_{r}^{*}$ in comparison to their counterparts modeled by the squirmer framework. Notably, this disparity is less pronounced than the divergence observed between squirmer and force-driven microrobots. Therefore, the conclusion that microrobots with active surface propulsion mechanisms are moderately more efficient in navigating through blood than their nonactive counterparts remains robust and unchanged. Additionally, as the value of *β* increases, $C_{r}^{*}$ for Janus squirmer microrobots shows a decrement, with a particularly sharp decline observed as *β* transitions from 1.0 to 1.5. Conversely, for squirmer microrobots, $C_{r}^{*}$ escalates with an increment in *β*, indicating a reduction in their navigational efficiency. Regarding *λ*, microrobots modeled by the squirmer and Janus models exhibit nearly the same values. These insights underscore the extensive versatility of the squirmer model, affirming its value as a foundational tool in the study of microrobot dynamics in blood. Simultaneously, the comparative analysis of the squirmer and Janus squirmer microrobot models also highlights the superior characteristics of the Janus squirmer microrobots, especially for the puller microrobots.

## Conclusion

In summary, we investigated the locomotion characteristics of surface-active and inert microrobots via three different models: force-driven, squirmer-type, and Janus squirmer microrobots. The coupling of the boundary element method (BEM) and FEM allowed for analyzing microrobot behavior in detail. We quantified their locomotion by calculating the relative resistance coefficient ($C_{r}^{*}$) and the lateral drift (*λ*) under varying conditions, including the Capillary number (*Ca*), hematocrit levels (*ϕ*), magnetic torque ($T_{m}^{*}$), and microrobot to RBC radius ratios (ϵ). Through comparative analysis, we delineated the behavior of the squirmer and Janus squirmer microrobots. This included a detailed examination of how the distinctive surface characteristics of each type of microrobot affect their propulsion mechanisms and efficacy in navigating through complex fluidic environments, which is crucial for potential biomedical applications. The main findings are as follows:

First, surface-active microrobots including squirmer and Janus squirmer microrobots are moderately more effective than inert microrobots in moving through blood, as they steer RBCs laterally and create the channel. Second, $C_{r}^{*}$ becomes more pronounced with increasing *ϕ* for surface and nonsurface-active microrobots. Third, applying magnetic torque reduces *λ* and $C_{r}^{*}$. However, *λ* and $C_{r}^{*}$ reach an approximate basin when $T_{m}^{*}$ ≥ 50. Fourth, microrobot to RBC radius ratio (ϵ) notably affects $C_{r}^{*}$. The squirmer microrobots exhibit nearly identical $C_{r}^{*}$ values when the sizes of the microrobot and RBCs are comparable. However, for considerably smaller microrobot sizes, puller microrobots exhibit enhanced $C_{r}^{*}$ than other squirmer microrobots. This difference can be attributed to the dominance of the nearest RBC in determining the behavior of small microrobots, while multiple RBCs extensively influence the behavior of large microrobots. Fifth, higher $C_{r}^{*}$ is observed in puller microrobots compared with pusher microrobots under high *Ca*; however, the trend is reversed under low *Ca*, owing to deformed RBCs.

Finally, our findings reveal that Janus squirmer microrobots exhibit a lower $C_{r}^{*}$ compared with squirmer microrobots, particularly at a *β* of 1.5, suggesting enhanced efficiency in certain conditions. This variance in performance can likely be attributed to the distinctive discontinuous slip velocity characteristic of Janus squirmer microrobots, which could be advantageous in environments where precise maneuverability and minimal disturbance to the surrounding medium are crucial. The distinct characteristics of Janus squirmer microrobots open avenues for targeted medical interventions, particularly in areas where gentle yet effective navigation is required.

While Janus microrobots exhibit promising efficiency in silico, their realization *in vivo* faces challenges (50). In real blood vessels and capillaries, the dynamic environment imposes significant challenges for surface-active microrobots. The high propulsion strength required to navigate the blood flow within vessels and capillaries necessitates significant advancements in harnessing endogenous fuels. This requirement significantly increases the complexity of implementing surface-driven propulsion in such environments. In contrast, driving microrobots using external forces, such as magnetic fields, remains a feasible and controllable strategy, offering a wider range of propulsion control and adaptability. Although some bioswimmers have demonstrated unique adaptations to blood flow, the practical application of these biological mechanisms to achieve functions similar to those of microrobots, such as drug delivery and noninvasive surgery, remains under exploration (51).

Overall, our study provides valuable insights into the locomotion characteristics of surface-active and nonsurface-active microrobots in blood. Although our study employed magnetic control as the navigation technique, the findings can be applied to electronic, optical, or acoustic navigation methods. The knowledge gleaned from this study provides a solid foundation for the design, advanced control strategies, and navigation techniques of microrobots with enhanced locomotion capabilities, enabling enhanced navigation through the circulatory system and interaction with biological entities. These advancements may revolutionize targeted drug delivery, minimally invasive surgeries, and other biomedical interventions.

## Methods

Simulations play an increasingly crucial role in robotics, enabling preliminary research into physical designs and control strategies before developing a real-world device (52). BEM–FEM coupling is one of the most accurate numerical methods for solving RBC deformation in the Stokes flow regime (29) because BEM incorporates the discontinuity of the hydrodynamic stress tensor across the RBC membrane. Our previous research has shown that the results obtained through BEM–FEM align closely with experimental results (30–32, 53). In this section, we explain our problem setting, basic equations, and numerical methods.

### Surface active microrobot: squirmer

We utilize the squirmer model to characterize the surface-active microrobot (15, 33, 54). Some active colloids self-propel in aqueous media by generating local gradients of concentration or electric potential via surface reactions. Flow around a phoretic microrobot can be split into bulk and interfacial regions around the particle (35). Under the assumption that phoretic effects are confined to the thin interfacial region, such effects can be represented as surface slip velocities. Therefore, a phoretic microrobot can be modeled as a squirmer. Some droplets also self-propel in aqueous media by generating Marangoni flow that can be attributed to interfacial energy gradients on the droplet interfaces (36). Thutupalli *et al.* (55) and Herminghaus *et al.* (56) reported that the surface slip velocities of self-propelled droplets were similar to the velocity of a squirmer. Moreover, ciliates have many hair-like cell organelles called cilia on the body’s surface. Ciliates swim by beating cilia, and the squirmer model can also represent ciliates swimming (14, 57, 58).

The squirmer is assumed to be spherical, neutrally buoyant, and non-Brownian, and to swim at a very small Reynolds number due to its small size. The squirmer’s surface is assumed to force the surrounding fluid tangentially, and these tangential motions are axisymmetric and time-independent. Based on Ishikawa *et al.* (59), the tangential surface speed $v_{s}$ on a squirmer is given as


(3) $$v_{s}=\frac{3U_{0}}{2}(\sin\;\psi +\beta \sin\;\psi \cos\;\psi ),$$

where $U_{0}$ is the swimming speed of an isolated squirmer, and *ψ* is the angle from the orientation vector e. The first term gives rise to swimming, while the second term controls the stress field. The parameter *β* can have either sign; a squirmer with β>0 is classified as a puller that generates thrust in the anterior side, β=0 represents a neutral microrobot, and β<0 designates a pusher that generates thrust in the posterior side (15). The velocity fields around them are shown in Fig. 1B, C, and D.

The Janus model represents a specialized iteration of the squirmer framework. By incorporating a sufficient number of squirming modes, the squirmer model can emulate the tangential surface speed profile characteristic of the configuration provided by the Janus model. The tangential surface speed $v_{s}$ on a Janus squirmer microrobot is given as (37)


(4) $$v_{s}=\left\{ \begin{array}{cc} \left(\frac{3}{2}+\beta \right)U_{0}\sin\;\psi , & \text{ for}\;\cos\;\psi \geq 0,\\ \left(\frac{3}{2}-\beta \right)U_{0}\sin\;\psi , & \text{ for}\;\cos\;\psi <0, \end{array}\right.$$

Figures 5A and S3 show $v_{s}$ for the squirmer and Janus squirmer microrobots with various *β*.

Additional torque is necessary to guide the locomotion of squirmer-type microrobots and prevent their random movement in RBC suspensions. Therefore, we introduce a dimensionless magnetic torque, $T^{*}$, given by


(5) $$T^{*}=\frac{4\pi \left|M\right|\left|B\right|a_{s}}{3\mu U_{0}}\sin(\theta )n_{T}=T_{m}^{*}\sin(\theta )n_{T},$$

where M represents the magnetization of the microrobot, aligned with the microrobot’s orientation e. B denotes the external magnetic field, and *θ* is the angle between M (or e) and B. $n_{T}$ is the unit vector associated with the magnetic torque. The maximum magnitude of $T^{*}$ is labeled $T_{m}^{*}$.

Another significant dimensionless number, ϵ, quantifies the size effect of the microrobot relative to that of the RBCs, which is defined as


(6) $$\epsilon =\frac{a_{s}}{a_{R}}.$$

Here, $a_{s}$ is the radius of the microrobot, and $a_{R}$ is the characteristic radius of an RBC defined as $3\sqrt{\frac{3V_{R}}{4\pi }}$, where $V_{R}$ is the volume of an RBC.

### Fluid mechanics of RBCs and the microrobot

Consider the locomotion of a force-driven or squirmer-type microrobot in an infinite suspension of blood cells, as shown in Fig. 1A. The microrobot is assumed to be a rigid sphere. The basic equations are explained here. Approximately 99% of the formed elements of blood are RBCs; hence, the infinite blood domain is modeled as *N* RBCs suspended in a triply periodic suspension. We assume that each RBC membrane is represented as a hyperelastic material, and internal and external RBC fluids have the same density *ρ* and viscosity *μ*. Due to the small size of RBCs and microrobots, the flow field inside and outside the RBCs can be simplified to Stokes flow. Under these conditions, the velocity at a specific point x in the fluid domain can be given by the boundary integral formulation (60)


(7) $$\begin{array}{rl} v(x) & =-\frac{1}{8\pi \mu }\sum_{n=1}^{N}\int J^{E}(x,y)\cdot q_{R}(y)dA_{R,n}(y)\\ & \;-\frac{1}{8\pi \mu }\int J^{E}(x,y)\cdot q_{s}(y)dA_{s}(y)\\ & \;+\frac{1}{8\pi \mu }\sum_{\;j=1}^{N_{F}}J^{E}(x,y_{\;j}^{G})\cdot F_{\mathrm{rep}}(y_{\;j}^{G}) \end{array}$$

where $q_{R}$ and $q_{s}$ are the force densities on the RBC membrane and microrobot surface, respectively. $A_{R}$ and $A_{s}$ represent RBC and microrobot surfaces, respectively. $J^{E}$ is Green’s function for the triply periodic lattice and is solved via the Ewald summation technique (61). The third term on the right of Eq. 7 represents the short-range repulsive force between RBC–RBC and RBC–microrobot. Hydrodynamic interactions alone cannot prevent collision of a squirmer to RBCs (62–64) and short-range repulsive forces $F_{\mathrm{rep}}$ must be introduced to avoid such collisions. The detailed form of $F_{\mathrm{rep}}$ is explained in *D—Numerical method*. $N_{F}$ is the number of Gauss points where repulsive forces are generated.

On the surface of a force-driven microrobot, the velocity satisfies the following boundary condition:


(8) $$v(x)=U+\Omega \wedge (x-x_{c}),\;x\in A_{s},$$

where U and Ω are translational and rotational velocities of the microrobot, respectively, and $x_{c}$ is the center of the sphere. On the surface of a squirmer-type microrobot, the velocity satisfies the following boundary condition:


(9) $$v(x)=U+\Omega \wedge (x-x_{c})+v_{s},\;x\in A_{s},$$

where $v_{s}$ is the surface squirming velocity. $v_{s}$ has only a tangential component given by Eq. 3. The velocity of the RBC membrane at point x is determined by the kinematic condition


(10) $$\frac{dx}{dt}=v(x),\;x\in A_{R},$$

### Solid mechanics of RBC membrane

RBC membrane is assumed to be an isotropic and hyperelastic material that follows the Skalak law (65) and Helfrich model (66). The method is identical to our previous research (67, 68).

The surface load $q_{R}$ in Eq. 7 consists of two components: the force due to in-plane membrane stretching $q_{s}$, the bending resistance $q_{b}$. The summation of two loads, $q_{R}=q_{s}+q_{b}$, which is utilized for coupling with fluid mechanics.

The surface deformation gradient tensor $D_{s}$ is given by


(11) $$dx=D_{s}\cdot dX,$$

where X and x are the membrane material points of the reference and deformed states, respectively. Local deformation of the RBC membrane is described by the Green–Lagrange strain tensor:


(12) $$E=\frac{1}{2}(D_{s}^{T}\cdot D_{s}-I_{s}),$$

where $I_{s}$ is the tangential projection operator. Two invariants of the in-plane strain tensor E are


(13) $$I_{1}=\lambda_{1}^{2}+\lambda_{2}^{2}-2,$$

(14) $$I_{2}=\lambda_{1}^{2}\lambda_{2}^{2}-1,$$

where $\lambda_{1}$ and $\lambda_{2}$ are the principal stretch ratios. The load due to in-plane stretching, $\left(q_{s}\right)$, is derived from the local equilibrium equation for the Cauchy tension, T, as:


(15) $$q_{s}=\nabla_{s}\cdot T,$$

where $\nabla_{s}$ is the surface gradient operator. The Cauchy stress tensor T is


(16) $$T=\frac{1}{S}D_{s}\cdot \frac{\partial w_{s}}{\partial E}\cdot D_{s}^{T},$$

where $S=\lambda_{1}\lambda_{2}$ is the area dilation ratio. Invoking the two-dimensional constitutive law of Skalak (65), the elastic strain energy per unit area $w_{s}$ can be expressed as


(17) $$w_{s}=\frac{G_{s}}{2}(I_{1}^{2}+2I_{1}-2I_{2}+CI_{2}^{2}),$$

where $G_{s}$ is the shear elastic modulus of RBC membrane, and *C* is the area dilation modulus. We set C=10 in this study to express the incompressibility of RBC membrane accurately (30).

In the context of $q_{s}$ solved by FEM, the weak form of the equilibrium condition for the RBC membrane is given by


(18) $$\int_{A}\hat{u}\cdot q_{s}\;dA\;=\int_{A}\hat{\epsilon }\;:\;T\;dA,$$

where $\hat{u}$ and $\hat{\epsilon }$ are the virtual displacement and strain, respectively.

Bending stiffness is characterized by the Helfrich model (66, 67), with the corresponding bending energy, $w_{b}$, is express as


(19) $$w_{b}=\frac{E_{b}}{2}\int_{A}\left(2H-c_{0}\right)\;dA,$$

where *H* is the mean curvature of the surface and $c_{0}$ is the spontaneous curvature. The bending modulus, $E_{b}$, is given by $E_{b}=0.05G_{s}a_{R}^{2}$. Assuming a symmetric membrane property, i.e. $c_{0}=0$, the bending energy can be converted to force density $q_{b}$ as


(20) $$q_{b}=-2E_{b}\left[\Delta_{s}H+2H\left(H^{2}-K\right)\right]n_{n},$$

where $\Delta_{s}$ is the Laplace-Beltrami operator on the surface and *K* is the Gaussian curvature, and $n_{n}$ is the normal vector of the node.

Since RBC deformation gets more pronounced with increasing viscous force, we introduce a dimensionless Capillary number *Ca*, representing the ratio of viscous force generated by the microrobot to the elastic force of the RBC membrane and defined as


(21) $$Ca=\frac{\mu U_{0}a_{s}}{G_{s}a_{R}},$$

where $U_{0}$ is the speed of the microrobot in an infinite Newtonian fluid with viscosity *μ*, devoid of any red blood cells.

### Numerical method

The numerical method developed by Foessel *et al.* (49, 69) was employed to couple the BEM for fluid mechanics with the FEM for solid mechanics. The number of RBCs in a unit domain is set as 44. The side length *L* of the unit computational domain is $L=9.03a_{R}∼12.27a_{R}$, depending on the hematocrit *ϕ*. The magnetic field is applied in the diagonal direction of the main domain. The explicit second-order Runge–Kutta method is conducted to update the position.

To discretize each RBC membrane, we utilized an unstructured triangular mesh generated through the recursive subdivision of an icosahedron. A fine mesh with a small element size is necessary to capture the velocity disruptions caused by near-field forces accurately. In this simulation, we used 1,280 triangular elements to discretize each RBC, ensuring sufficient resolution. The integrals in Eq. 7 are calculated using Gauss points on the elements.

To handle the singularity of 1/r in Eq. 7, coordinate transformation to cylindrical coordinates is locally employed (70). For the velocity caused by far-field forces, the adaptive mesh refinement method can achieve a coarser representation with high accuracy and extensively reduce the time cost of simulations. In performing the Ewald summation, we employed the multipole expansion technique (71, 72) to calculate far-field fluid dynamics efficiently.

The short-range repulsive force $F_{\mathrm{rep}}$ is generated between Gauss points on triangular boundary elements of RBCs and microrobot. $F_{\mathrm{rep}}$ at a Gauss point $y_{j}^{G}$ is given as


(22) $$F_{\mathrm{rep}}(y_{\;j}^{G})=\left\{ \begin{array}{cc} \sum_{i}^{m}k_{c}d_{G}\frac{y_{j}^{G}-x_{i}^{G}}{\left|y_{j}^{G}-x_{i}^{G}\right|}\; & d_{G}<0.15a_{R}\\ 0 & d_{G}\geq 0.15a_{R}, \end{array}\right.$$

where $k_{c}$ is the spring constant. $d_{G}$ is the distance between two Gauss points given by $d_{G}=|y_{j}^{G}-x_{i}^{G}|$, where $x_{i}^{G}$ are the nearby Gauss points of distinct RBCs or the microrobot. In this study, we set $k_{c}$ to $0.08G_{s}$ and the threshold distance to $0.15a_{R}$ to avoid overlapping of surfaces. All nomenclature is summarized in Table 1.

## Supplementary Material

pgae463_Supplementary_Data

## Data Availability

All study data are included in the article and Supplementary Material, Appendix.
