# Supplementary material for: Surface-active microrobots can propel through blood faster than inert microrobots
Source: PNAS Nexus. 2024 Oct 15;3(10):pgae463. doi: 10.1093/pnasnexus/pgae463 (PMC11518928; doi:10.1093/pnasnexus/pgae463)
Supplement: pgae463_Supplementary_Data [file pgae463_supplementary_data.zip › PNASNEXUS-PNASNEXUS-2024-00644-TR-s04.pdf]

Supporting Information for

Surface-Active Microrobots can Propel through Blood Faster than Inert Microrobots

Chenjun Wu, Toshihiro Omori and Takuji Ishikawa

Corresponding Author Takuji Ishikawa
E-mail: t.ishikawa@tohoku.ac.jp

This PDF file includes:

Supporting text
Figs. S1 to S5
Legends for Movies S1 to S3
SI References

Other supporting materials for this manuscript include the following:

Movies S1 to S3

Supporting Information Text

S1 - EFFECT OF HEMATOCRIT

We tuned the hematocrit (ϕ) of red blood cell (RBC) suspensions to assess their impact on the resistance coefficient (C_r^*) and lateral drift (λ) of various microrobots. Adhering to the definitions provided in the main text, Ca, ϵ, and T_m^* are held constant at 0.5, 0.8, and 10, respectively. C_r^* increases with rising ϕ for all microrobots, as shown in Fig. S1A. The puller microrobot exhibits lower C_r^* values than the puller and neutral microrobots. The main reason is that the surface velocity of the puller microrobot steers the red blood cells laterally, allowing them to propel themselves into the hole they are digging. The minimum C_r^* for the force-driven microrobot extensively exceeds the maximum C_r^* for the squirmer-type microrobots, providing compelling evidence of the superiority of squirmer-type microrobots.

Figure S1B. shows λ for the microrobots under varying ϕ. Variation in ϕ triggered fluctuations in λ for the squirmer-type microrobots. At $\phi = 25\%$, λ of neutral-type microrobot is approximately 0.1, exhibiting a disadvantage. In this scenario, implementing advanced location, navigation, and control technology for microrobots is imperative, especially in *in vivo* applications (1). The force-driven microrobot demonstrates a significant competitive edge over the squirmer-type microrobots in terms of λ, attributed to the lower λ values.

S2 - VELOCITY MAGNITUDE OF THE SQUIRMER MICROROBOTS UNDER VARIOUS MAGNETIC TORQUE

Adhering to the definitions provided in the main text, where U_d and U_r represent the velocities of the microrobot in the diagonal (along magnetic field direction) and corresponding perpendicular directions, respectively, we compute the average speed of the microrobot as $\langle|\boldsymbol{U}|\rangle$, under various magnetic torques. The speed of the microrobot $|\boldsymbol{U}|$ is defined as

$$|\boldsymbol{U}| = \sqrt{U_d^2 + U_r^2} \qquad \text{[S1]}$$

Capillary number (Ca), the size ratio between the microrobots and RBCs (ϵ), and hematocrit (ϕ) are held constant at 0.5, 0.8, and 25%, respectively. The results are illustrated in Fig. S2. The nearly same $|\boldsymbol{U}|$ values are found for squirmer microrobots across a spectrum of magnetic torque levels, suggesting that magnetic torque plays a role in enhancing the velocity of microrobots along the magnetic field direction and simultaneously constraining their velocity perpendicular to the magnetic field. Conventional force-driven and force-magnetic-torque-driven (FMT) microrobots also show the nearly same $|\boldsymbol{U}|$ values when T_m^* is greater than 1, although their $|\boldsymbol{U}|$ values are lower than those of squirmer microrobots.

S3 - Mean squared angular spread $\langle\theta^2\rangle$ of the squirmer microrobots

We calculate their mean squared angular spread ($\langle\theta^2\rangle$) under various values of T_m^*. $\langle\theta^2\rangle$ is defined as

$$\langle\theta^2\rangle = \frac{1}{NM}\sum_{i=1}^{N}\sum_{m=0}^{M-1}\theta_{i,m}^2 \qquad \text{[S2]}$$

where N is the number of simulations in a certain setting ($N = 5$). M represents the total number of discrete time intervals. The results are detailed in Fig. S3. The squirmer microrobots experience a decrease in $\langle\theta^2\rangle$ with increasing T_m^*. Under low T_m^* ($T_m^* = 1.0$), a distinct $\langle\theta^2\rangle$ is observed among

the squirmer microrobots. When T_m^* is larger than 50, all squirmer microrobots show nearly the same $\langle \theta^2 \rangle$.

S4 - TIME-AVERAGED MAX ISOTROPIC MEMBRANE TENSION

During *in vivo* operation, microrobots generate forces on adjacent red blood cells (RBCs), potentially causing hemolysis (2). To evaluate this risk and gain deeper insights into the microrobots' mechanical interactions with RBCs, we quantified the time-averaged maximum isotropic membrane tension, $\langle \tau_{\mathrm{p}}^{\max} \rangle$ on all RBC membranes under $\phi = 25\%$, $Ca = 1.1$, and $\epsilon = 0.8$. $\langle \tau_{\mathrm{p}}^{\max} \rangle$ is expressed as

$$\langle \tau_{\mathrm{p}}^{\max} \rangle = \frac{1}{M} \sum_{i=0}^{M-1} \tau_{\mathrm{p},i}^{\max} \qquad \text{[S3]}$$

where $\tau_{\mathrm{p},i}^{\max}$ is the maximum τ_{p} at each time point from five independent trials. M represents the total number of discrete time intervals. This metric allowed us to characterize the deformation of RBCs under these conditions (2). Isotropic membrane tension on the RBC membranes, τ_{p}, is defined as:

$$\tau_{\mathrm{p}} = \frac{\tau_1 + \tau_2}{2}. \qquad \text{[S4]}$$

where principal tensions in the membrane τ_1 and τ_2 ($\tau_1 > \tau_2$) of the Skalak law are given by (3)

$$\begin{aligned} \tau_1 &= \frac{G_s \lambda_1}{\lambda_2} \left[\lambda_1^2 - 1 + C \lambda_2^2 (\lambda_1^2 \lambda_2^2 - 1) \right] \\ \tau_2 &= \frac{G_s \lambda_2}{\lambda_1} \left[\lambda_2^2 - 1 + C \lambda_1^2 (\lambda_1^2 \lambda_2^2 - 1) \right] \end{aligned} \qquad \text{[S5]}$$

where $\lambda 1$ and $\lambda 2$ are the two principal in-plane stretch ratios, and C is a dimensionless material coefficient that measures the resistance to area dilation. C is set at 10, consistent with the value used in the main text. G_{s} is the shear elastic modulus of the RBC membrane. The results are illustrated in Fig. S4. The puller microrobot exhibits the highest $\langle \tau_{\mathrm{p}}^{\max} \rangle$. As noted in the main text, it also shows the highest C_{r}^* among squirmer microrobots in the same conditions. This suggests that the puller microrobot not only moves the slowest among the squirmer microrobots but also poses a heightened risk of hemolysis, making it unsuitable for operation at high Ca. In contrast, the pusher microrobot shows the lowest $\langle \tau_{\mathrm{p}}^{\max} \rangle$ among the squirmer microrobots. The force-driven microrobot exhibits the lowest $\langle \tau_{\mathrm{p}}^{\max} \rangle$ across all microrobots, suggesting its potential as a safer alternative with minimal risk to surrounding red blood cells despite its high C_{r}^* .

Table S1: Types of Microrobots in This Study

<table>
<tr><th colspan="5">Surface-active microrobots (Squirmer microrobots)</th><th colspan="2">Inert microrobots</th></tr>
<tr><th colspan="3">Squirmer retaining terms up to the second order (abbreviated as squirmer)</th><th colspan="2">Janus squirmer</th><th rowspan="2">Force driven microrobot</th><th rowspan="2">FMT microrobot</th></tr>
<tr><th>Puller $\beta = 1$</th><th>Neutral $\beta = 0$</th><th>Pusher $\beta = -1$</th><th>Puller $\beta = 1$</th><th>Pusher $\beta = -1$</th></tr>
</table>

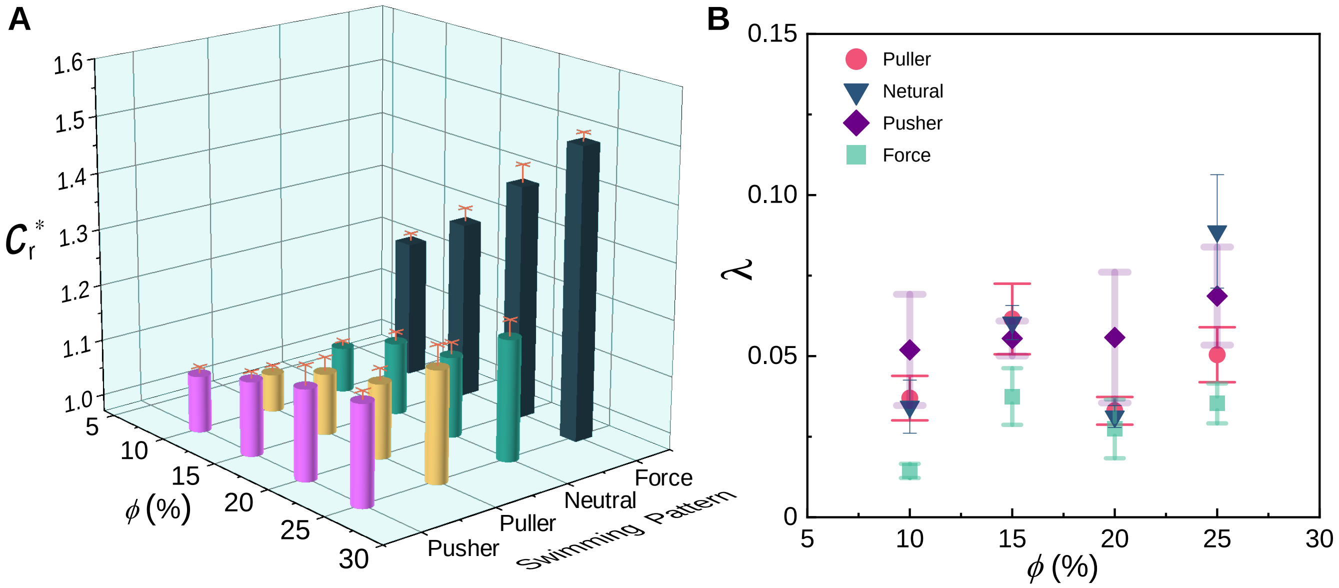

Fig. S1. Effect of hematocrit (ϕ) on the relative resistance coefficient **(A)** and lateral drift **(B)** of the various microrobots. T_m^*, Ca, and ϵ are set to 10, 0.5, and 0.8, respectively. Each data point for microrobot locomotion shows the means and standard errors of five independent trials.

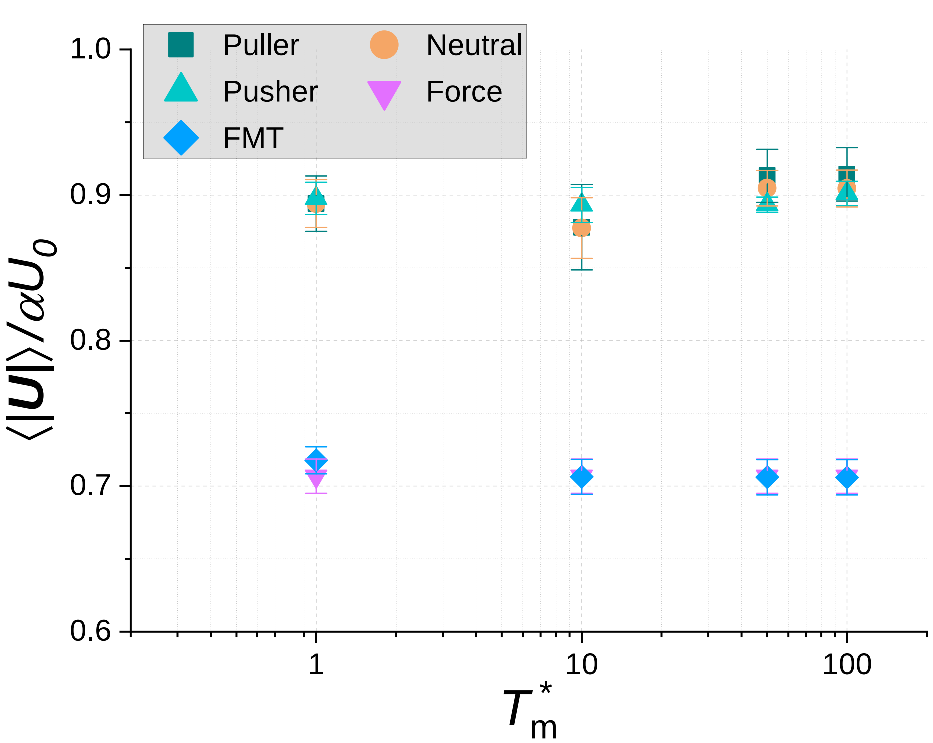

Fig. S2. The average velocity magnitude of microrobots under various magnetic torques. ϕ, Ca, and ϵ are set to 25%, 0.5, and 0.8, respectively. Each data point for microrobot locomotion shows the means and standard errors of five independent trials. The α values for squirmer microrobots and force-driven microrobots (both conventional and FMT microrobots) are set at 1.0 and 0.7517 (Table 2), respectively.

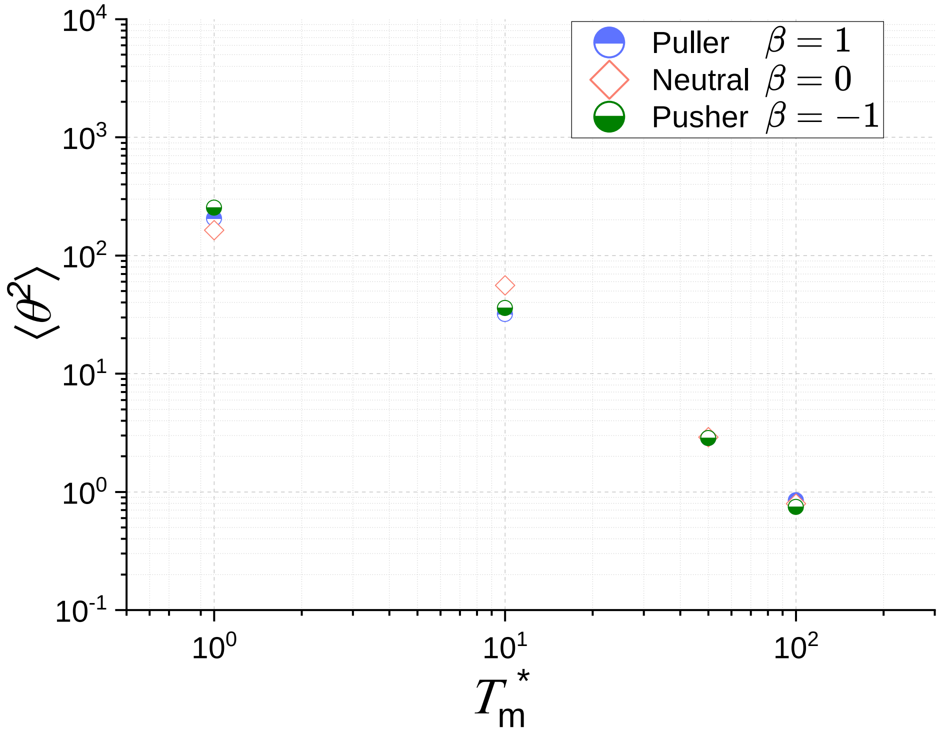

Fig. S3. Mean squared angular spread $\langle\theta^2\rangle$ of the squirmer microrobots under various magnetic torques (T_m^*). ϕ, Ca, and ϵ are set to 25%, 0.5, and 0.8, respectively.

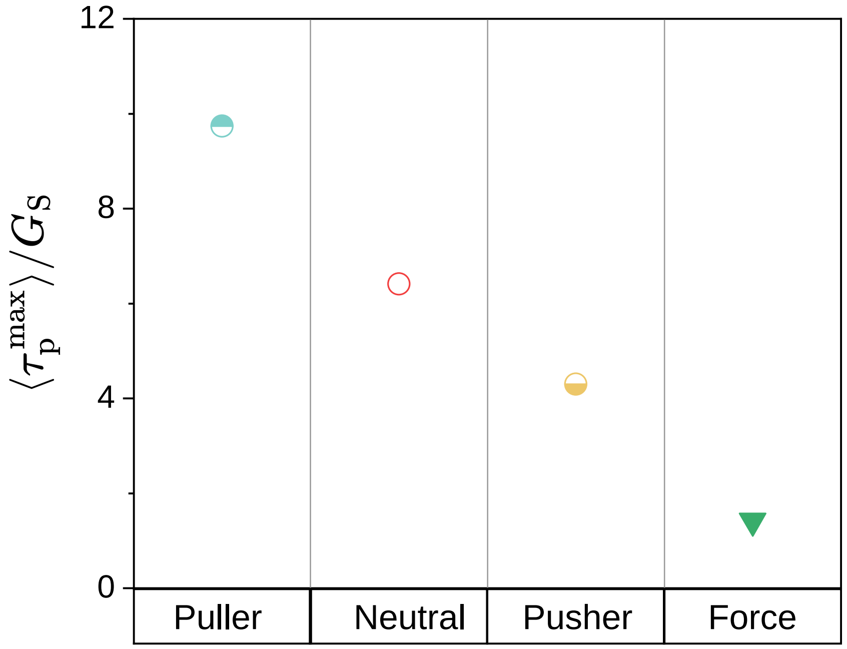

Fig. S4. Average maximum isotropic membrane tension on the membrane of RBCs. ϕ, Ca, and ϵ are set to 25%, 1.1, and 0.8, respectively.

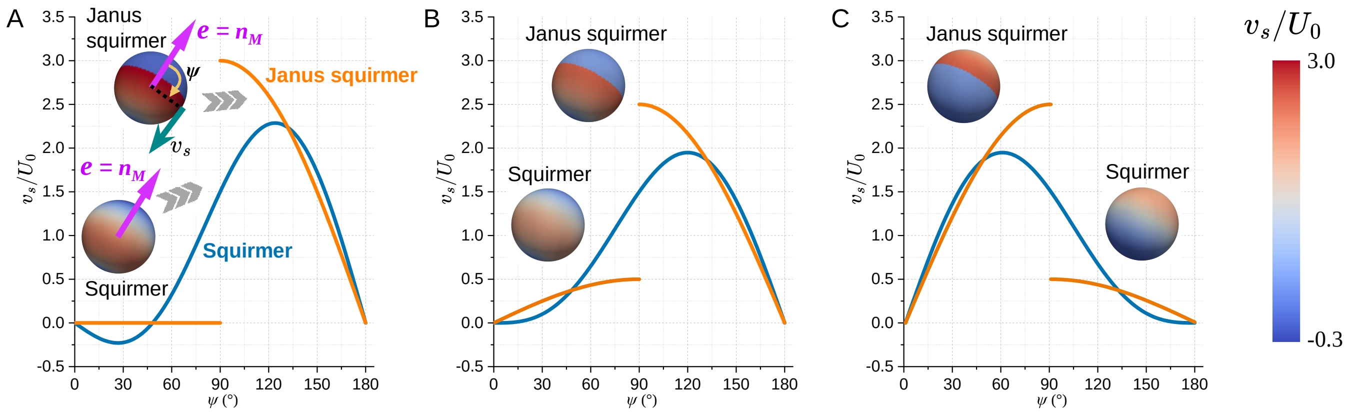

Fig. S5. Surface velocity profiles for Janus squirmer and squirmer microrobots: Pusher-type **(A,B)** with β = -1.5 **(A)** and β = -1.0 **(B)**, and Puller-type with β = 1.0 **(C)**. The orientation (e) and magnetization direction ($\boldsymbol{n}_{\mathrm{M}}$) of the squirmer and Janus squirmer microrobots are identical. Surface velocity for squirmer and Janus squirmer microrobots are illustrated in Eq. (3) and Eq. (4) in the main text.

Movie S1. Locomotion of Neutral-type squirmer microrobot in blood, characterized by Hematocrit (ϕ) of 25%, Capillary number (Ca) of 0.5, and radius ratio (ϵ) of 0.8 between the microrobot and a red blood cell under a dimensionless maximum magnetic torque T^*_{m} of 10. The dynamic interplay between the microrobot and red blood cells is vividly captured and highlighted. The footage tracks the center of the microrobot.

Movie S2. Trajectories of pusher (pink), neutral (dark green), puller(yellow), and force-driven (blue) microrobots in blood characterized by Hematocrit (ϕ) of 25%, Capillary number (Ca) of 0.5, and radius ratio (ϵ) of 0.8 between the microrobot and a red blood cell under a dimensionless maximum magnetic torque T^*_{m} of 10. The red blood cells are omitted for clear display. While each trajectory was computed in separate simulations, they are concurrently displayed within a singular visual framework to facilitate direct comparative analysis. The tetragonal cone structure represents the orientation (e) of the squirmer microrobots and is not a structural component of the microrobots.

Movie S3. Interaction between a single puller microrobot with a red blood cell in the lab frame. The angle (θ_c) between the normal vector of the RBC plane ($n_{\mathbf{RBC}}$) and x-axis is set at $15°$. In the upper segment, the puller microrobot with a size ratio (ϵ) of 0.2 interacts with a red blood cell. In the lower segment, the puller microrobot with $\epsilon = 1.0$ is shown encountering another red blood cell.

References

1. F Ongaro, S Pane, S Scheggi, S Misra, Design of an electromagnetic setup for independent three-dimensional control of pairs of identical and nonidentical microrobots. *IEEE Trans. Robot.* **35**, 174–183 (2018).
2. M Sitti, Miniature soft robots—road to the clinic. *Nat. Rev. Mater.* **3**, 74–75 (2018).
3. R Skalak, A Tozeren, R Zarda, S Chien, Strain energy function of red blood cell membranes. *Biophys. J.* **13**, 245–264 (1973).
